# Identification of the antiepileptic racetam binding site in the synaptic vesicle protein 2A by molecular dynamics and docking simulations

**DOI:** 10.3389/fncel.2015.00125

**Published:** 2015-04-10

**Authors:** José Correa-Basurto, Roberto I. Cuevas-Hernández, Bryan V. Phillips-Farfán, Marlet Martínez-Archundia, Antonio Romo-Mancillas, Gema L. Ramírez-Salinas, Óscar A. Pérez-González, José Trujillo-Ferrara, Julieta G. Mendoza-Torreblanca

**Affiliations:** ^1^Laboratorio de Modelado Molecular y Diseño de fármacos, Departamento de Bioquímica de la Escuela Superior de Medicina del Instituto Politécnico Nacional, México CityMexico; ^2^Laboratorio de Nutrición Experimental, Laboratorio de Oncología Experimental and Laboratorio de Neuroquímica, Instituto Nacional de Pediatría, México CityMexico; ^3^División de Estudios de Posgrado, Facultad de Química, Universidad Autónoma de Querétaro, Santiago de QuerétaroMexico

**Keywords:** SV2A, epilepsy, levetiracetam, brivaracetam, seletracetam

## Abstract

Synaptic vesicle protein 2A (SV2A) is an integral membrane protein necessary for the proper function of the central nervous system and is associated to the physiopathology of epilepsy. SV2A is the molecular target of the anti-epileptic drug levetiracetam and its racetam analogs. The racetam binding site in SV2A and the non-covalent interactions between racetams and SV2A are currently unknown; therefore, an *in silico* study was performed to explore these issues. Since SV2A has not been structurally characterized with X-ray crystallography or nuclear magnetic resonance, a three-dimensional (3D) model was built. The model was refined by performing a molecular dynamics simulation (MDS) and the interactions of SV2A with the racetams were determined by docking studies. A reliable 3D model of SV2A was obtained; it reached structural equilibrium during the last 15 ns of the MDS (50 ns) with remaining structural motions in the N-terminus and long cytoplasmic loop. The docking studies revealed that hydrophobic interactions and hydrogen bonds participate importantly in ligand recognition within the binding site. Residues T456, S665, W666, D670 and L689 were important for racetam binding within the trans-membrane hydrophilic core of SV2A. Identifying the racetam binding site within SV2A should facilitate the synthesis of suitable radio-ligands to study treatment response and possibly epilepsy progression.

## Introduction

Epilepsy is the most common chronic brain disorder that affects people of all ages. More than 50 million people worldwide have epilepsy (WHO, 2014). It is characterized by recurrent seizures, which may vary from a brief lapse of attention or muscle jerks, to severe and prolonged convulsions (WHO, 2014). In most patients with epilepsy, seizures respond to available medications. However, a significant number of patients -especially in the setting of medical-intractable epilepsies- may experience different degrees of memory or cognitive impairment, behavioral abnormalities or psychiatric symptoms, which limit their daily functioning. As a result, in many patients, epilepsy may resemble a neurodegenerative disease (Ono and Galanopoulou, 2012). Epileptic seizures and/or epileptogenesis may functionally alter brain regions involved in cognitive processing, contributing to the progressive nature of epilepsy; additionally neurodegenerative cellular mechanisms may also participate. To cure epilepsy, both epileptogenesis and the associated neuro-degeneration have to be stopped and, if possible, reversed. This will require early detection through biomarkers that can reliably predict disease progression (Ono and Galanopoulou, 2012).

Synaptic vesicle protein 2A is an integral membrane protein present on all synaptic vesicles, being nearly ubiquitous in the CNS (Lynch et al., 2004). The existing evidence suggests that SV2A is critical for the proper function of the CNS. It regulates vesicle exocytosis by modulating the concentration of pre-synaptic calcium (Janz et al., 1999; Chang and Sudhof, 2009; Wan et al., 2010); maybe by binding to synaptotagmin 1 (Schivell et al., 1996, 2005; Yao et al., 2010), a calcium sensor protein implicated in synaptic vesicle exocytosis. In addition, it has been proposed that SV2A may control the release of neurotransmitters as a gel matrix or could transport ions, such as chloride or calcium (Bajjalieh et al., 1994; Janz et al., 1999; Reigada et al., 2003; Mendoza-Torreblanca et al., 2013). The SV2A protein has been associated to the physiopathology of epilepsy; newborn mice lacking SV2A experience severe seizures and die within 3 weeks, suggesting multiple neural alterations (Crowder et al., 1999; Janz et al., 1999). Moreover, SV2A protein expression is significantly reduced in brain tissue obtained from epileptic patients and in rats during epileptogenesis, suggesting that decreased SV2A contributes to the progression of epilepsy (Feng et al., 2009; Toering et al., 2009; van Vliet et al., 2009). SV2A is the molecular target of the second-generation antiepileptic drug LEV and its structural analogs BRIV, SEL and UCB-30889 (Gillard et al., 2003; Lynch et al., 2004; Frycia et al., 2010). Although LEV may acutely modulate ion channels and other targets; chronically, LEV may lead to decreased transmitter release predominantly by binding to SV2A (Surges et al., 2008; Crepeau and Treiman, 2010; Lyseng-Williamson, 2011; Deshpande and Delorenzo, 2014).

Knowing the *in vivo* expression pattern of SV2A in epilepsy patients may be essential for evaluating increased epileptogenicity and thus disease progression (van Vliet et al., 2009). Moreover, SV2A expression could also be useful as a biomarker for treatment response (de Groot et al., 2011). For this purpose, sensitive neuroimaging methods such as PET may enhance our ability to detect SV2A expression (Ono and Galanopoulou, 2012). Because PET requires the use of a radio-ligand to label the protein target, as a first approximation to synthesize a radio-ligand with a high affinity and specificity, we investigated the specific binding site(s) for racetams in SV2A and the residues involved in their interaction. Therefore, an *in silico* study was performed to identify the binding site for LEV and other racetams within SV2A. Since SV2A has not been structurally characterized with X-ray crystallography or nuclear magnetic resonance, it was necessary to generate and validate a 3D model of this protein. This model was refined by a MDS and docking studies were performed to decipher the interactions between the racetams and SV2A.

## Materials and Methods

### SV2A Modeling

The SV2A protein sequence was retrieved from the National Center for Biotechnology Information (NP_476558.2) and UniProt (Q02563). There was no difference between these databases; the protein sequence was thus used as an input for the I-Tasser server (Roy et al., 2010); which provided a cluster of five 3D models for this protein. The Rampage server was used to obtain the Ramachandran plot, which shows the φ and ψ torsion angles for all protein residues. Additionally, the CPHmodels-3.2 and 3D-JIGSAWv3.0 servers were used to confirm the reproducibility of the SV2A 3D model provided by I-Tasser.

### System Preparation for Molecular Dynamics Simulation

The model of the SV2A protein was inserted into a pre-equilibrated membrane consisting of POPC molecules. The resulting system was then placed in a hexagonal prism shaped box with its symmetry axis, which was perpendicular to the plane of the bilayer membrane, in the *z* direction. The system was then solvated using simple point charge model water molecules. The geometry and size of the simulation box were carefully selected to reduce to a minimum the number of water molecules and thus decrease the computation time. In order to remove solvent molecules accidentally introduced in the hydrophobic region of the lipid bilayer, 19 Na^+^ ions were added to neutralize the whole system.

The simulation box contained one molecule of SV2A, 342 POPC lipids, 47,671 water molecules and 19 ions, resulting in a total of 172,298 atoms. The OPLS-AA force field was used, it has been used to simulate and describe several TM proteins with good results (Tieleman et al., 2006). The simulation was done by applying the half-𝜀 double-pair list method to ensure compatibility between the Berger united atom parameters, used for lipids (Lindahl and Edholm, 2000; Chakrabarti et al., 2010), with the OPLS-AA force field employed for protein and ions (Jorgensen et al., 1996; Kaminski et al., 2001).

### Molecular Dynamics Simulation

The system in its entirety was minimized for 500 steps using the *steepest descent* optimization algorithm prior to generating the MDS trajectory. Solvent–protein and lipid–protein contacts were optimized by a simulation lasting 5 ns, with harmonic constraints in the heavy atoms of the protein (all, except for hydrogen). The force constants of these positional restraints were sequentially reduced. Afterward, an unrestrained trajectory lasting 50 ns was generated. The simulation was performed using a 2 fs time step. The pressure was set at 1 bar using a semi-isotropic Parrinello and Rahman (1981) barostat (Nosé and Klein, 1983) to allow independent modification of the box dimensions in the *XY* plane and in the *Z* dimension. The temperature was kept constant at 310 K by a Nosé (1984) and Hoover (1985) thermostat. The linear constraint solver algorithm was employed to remove bond vibrations (Hess et al., 1997). The particle mesh Ewald method (Darden et al., 1993; Essmann et al., 1995) coupled to periodic boundary conditions was used to simulate long-range electrostatic interactions using a direct space cutoff of 1.2 nm and a grid spacing of 0.15 nm. Because periodic boundary conditions can produce periodic artifacts, especially if combined with Ewald methods (Essmann et al., 1995), the minimum distance between protein molecules in adjacent boxes was calculated as a function of time. The simulation box size was adequate for the system, based on the cutoff used for electrostatic and short range contributions to the potential function. The van der Waals interactions were computed using periodic boundary conditions coupled to a spherical cutoff of 1.2 nm. The MDS was performed using GROMACS 4.6.1 (Hess et al., 2008). Additionally, a replicate MDS was carried out in order to confirm the results of the first MDS.

### Docking Studies

Docking and MDS simulations were combined by screening different SV2A conformers. LEV, BRIV, SEL and UCB-30889 were the selected racetam ligands. The criteria to include these ligands were as follows: LEV specifically binds to SV2A (Lynch et al., 2004) and is the lead anti-convulsive racetam. BRIV, SEL and UCB-30889 also bind to SV2A, but with higher affinity than LEV (Gillard et al., 2003, 2011; Matagne et al., 2009). BRIV and SEL have been the two leading candidates to replace LEV, since they were more potent and effective in animals model studies (Kaminski et al., 2012). Finally, UCB-30889 has been extensively used to characterize the binding properties of SV2A (Gillard et al., 2003; Lambeng et al., 2005; Shi et al., 2011). Interactions between the racetam ligands and nine SV2A conformers corresponding to every 2 ns during the last 16 ns of the MDS trajectory (when the system reached equilibrium) were simulated. In order to obtain the minimum energy conformation for each ligand structure, they were pre-optimized with AM1 (a semi-empirical method) and then optimized using the B3LYP/6-31G(d) basis set, both implemented in Gaussian 03 (Frisch et al., 1998). Dockings were performed using AutoDock 4.0.1 and AutoDock Tools 1.5.6 (Morris et al., 2008).

Before starting the docking simulations, hydrogen atoms were added to the polar atoms (considering a 7.4 pH value) and the Kollman charges were assigned for all atoms in the receptor. All rotating bonds, torsional degrees of freedom, atomic partial charges and non-polar hydrogens of the ligands were assigned. Then the ligands were docked onto the SV2A protein using a 70 Å × 70 Å × 70 Å grid box which covered all putative residues involved in racetam recognition according to the literature (Shi et al., 2011). A grid spacing of 0.375 Å was used under the hybrid Lamarckian Genetic Algorithm with an initial population of 100 randomly placed individuals and 1 × 10^7^ as the maximum number of energy evaluations. All other docking parameters remained at their default settings. The resulting docked orientations within a root mean square deviation (RMSD) of 0.5 Å were clustered together. The lowest energy cluster for each compound, returned by AutoDock, was used for further analysis. The script and files used were prepared with AutoDock Tools 1.5.6 and this software was also used to visualize all the ligand–protein complexes together with VMD 1.9.1 (Humphrey et al., 1996). All the artwork was created using Gnuplot 4.6, VMD 1.8, Pymol 1.5 and Photoshop CS5 Extended 12.

## Results

### SV2A Modeling

**FIGURE 1:**
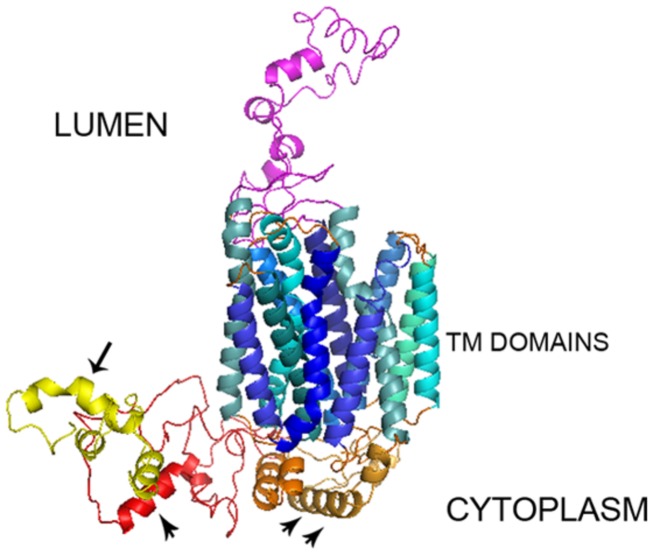
**3D SV2A model.** The I-Tasser server yielded a model of SV2A with 742 amino acids distributed in 12 TM domains consisting of α-helices, an undefined intra-luminal domain and two cytoplasmic domains: a relatively large N-terminus with a synaptotagmin 1 binding site (arrow) plus an ATP binding motif (arrow head) and a large loop between TM domains 6–7 with another ATP binding motif (two arrows heads).

After sending the SV2A protein sequence (FASTA format) to the I-Tasser server, it returned five possible 3D models with 12 α-helical TM segments, two long loops and a long cytoplasmic N-terminus. The *C*-score was between -2.84 to -3.02, the TM-score was 0.39 ± 0.13 and the RMSD was 15.4 ± 3.4 Å. The Ramachandran plot showed φ and ψ angles of 94.7–96.2 % in the favored-allowed regions of the SV2A models. In four of the models, the TM region was invaded importantly by the N-terminus. They were discarded for this reason and the model with an N-terminus separate from the TM region was selected (model 3; **Figure 1**). Moreover, the CPHmodels-3.2 and 3D-JIGSAWv3.0 servers were used to build 3D models of SV2A to confirm reproducibility. Both servers generated a structure quite similar to the I-Tasser model, predicting 12 TM regions.

### Molecular Dynamics Simulations

**FIGURE 2:**
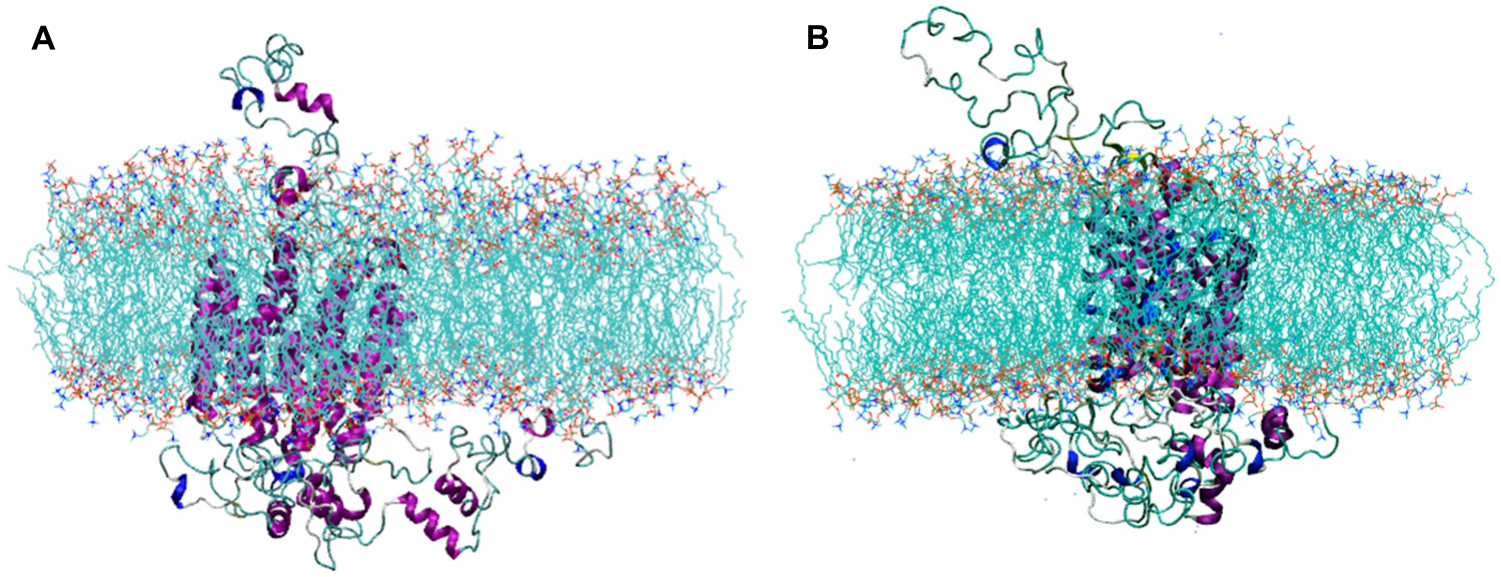
**Views of the simulation system, **(A)** after minimization and **(B)** after the MDS.** Notice that SV2A rearranges within the lipid bilayer.

The selected SV2A model was inserted into a POPC membrane and a MDS was performed to obtain a highly reliable 3D structure. **Figure 2** shows the simulation system after structural minimization (**Figure 2A**) and after 50 ns of MDS (**Figure 2B**). Notice the rearrangement of the SV2A protein, which indicates that conformational changes occur in the extra and intra-membrane domains, being greatest in loop structure components. The MDS allowed SV2A to re-accommodate within the membrane region, producing a more reliable system to perform docking studies of known ligands.

**FIGURE 3:**
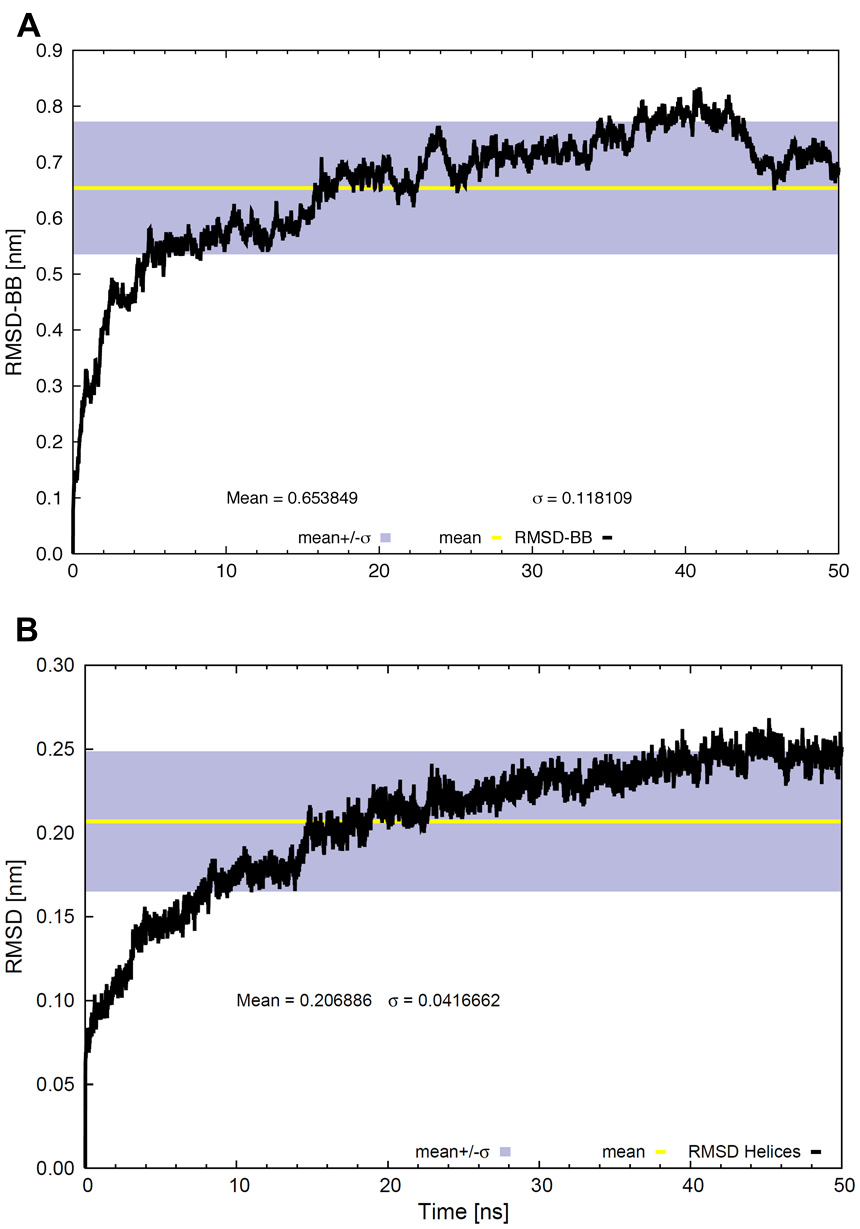
**Root mean square deviation values for **(A)** the whole backbone and **(B)** the backbone of the TM region as a function of time.** The gray bands correspond to the SD and the line within them represents the average value.

**FIGURE 4:**
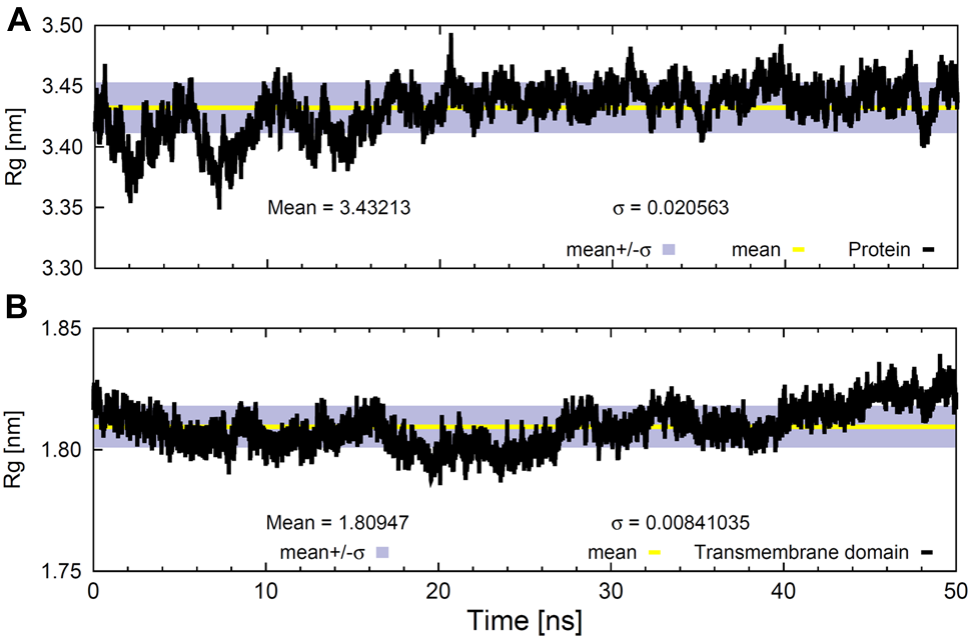
**Radius of gyration values for **(A)** the whole backbone and **(B)** the backbone of the TM region as a function of time.** The gray bands correspond to the SD and the line within them represents the average value.

**Figure 3** shows the RMSD values for α-carbons of the whole backbone (**Figure 3A**) or α-carbons of the TM backbone helical domains (**Figure 3B**). RMSD values typically help to visualize global structural changes to determine protein stability. The lower RMSD values (0.21 ± 0.04 nm) of the TM helical domains (**Figure 3B**), compared to the whole backbone (**Figure 3A**), indicate strong conformational changes in extra-membrane regions. The RMSD slope became modest at ∼20 ns and tended to zero beyond ∼30–35 ns. The RMSD values suggest that the system reaches equilibrium, as confirmed by the Rg values (**Figure 4**).

The Rg of the whole protein backbone did not show a clear trend throughout the 50 ns trajectory (**Figure 4A**); it oscillated around an average value of 3.43 ± 0.02 nm. However, the variation was much lower when only the backbone atoms of the TM helical domain were considered (**Figure 4B**), although the Rg values for this region tended to increase slightly. This could be due to the fact that SV2A is an integral membrane protein with fairly stable TM domains and flexible extra-membrane portions.

**FIGURE 5:**
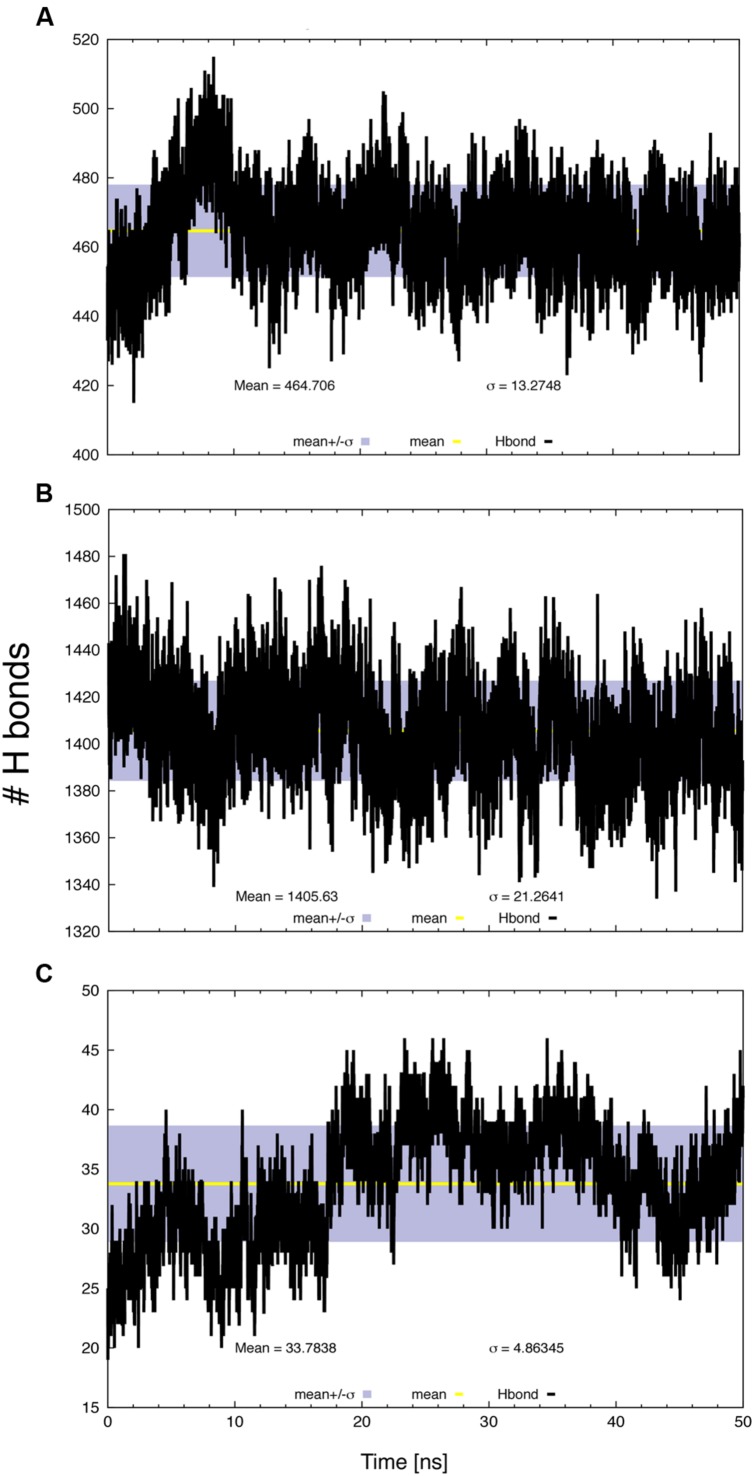
**Number of H-bonds as a function of time, **(A)** protein intra-molecular, **(B)** protein–water and **(C)** protein–lipid.** The gray bands correspond to the SD and the line within them represents the average value.

The number of intra-molecular (**Figure 5A**), protein–water (**Figure 5B**) and protein–lipid (**Figure 5C**) H-bonds was determined as a function of time, based on geometrical criteria (donor–acceptor distance cutoff <3.5 Å and donor-H-acceptor angle <30°). Changes in inter-helical hydrogen bonding are associated with the conformational dynamics of membrane proteins (Bondar and White, 2012). Most H-bonds corresponded to protein–solvent interactions, while only a few were formed between SV2A and the lipid heads. There was significant variation in the number of intra-molecular H-bonds that may correspond to a conformational change; however, this number reached an equilibrium value during the last part of the trajectory.

**FIGURE 6:**
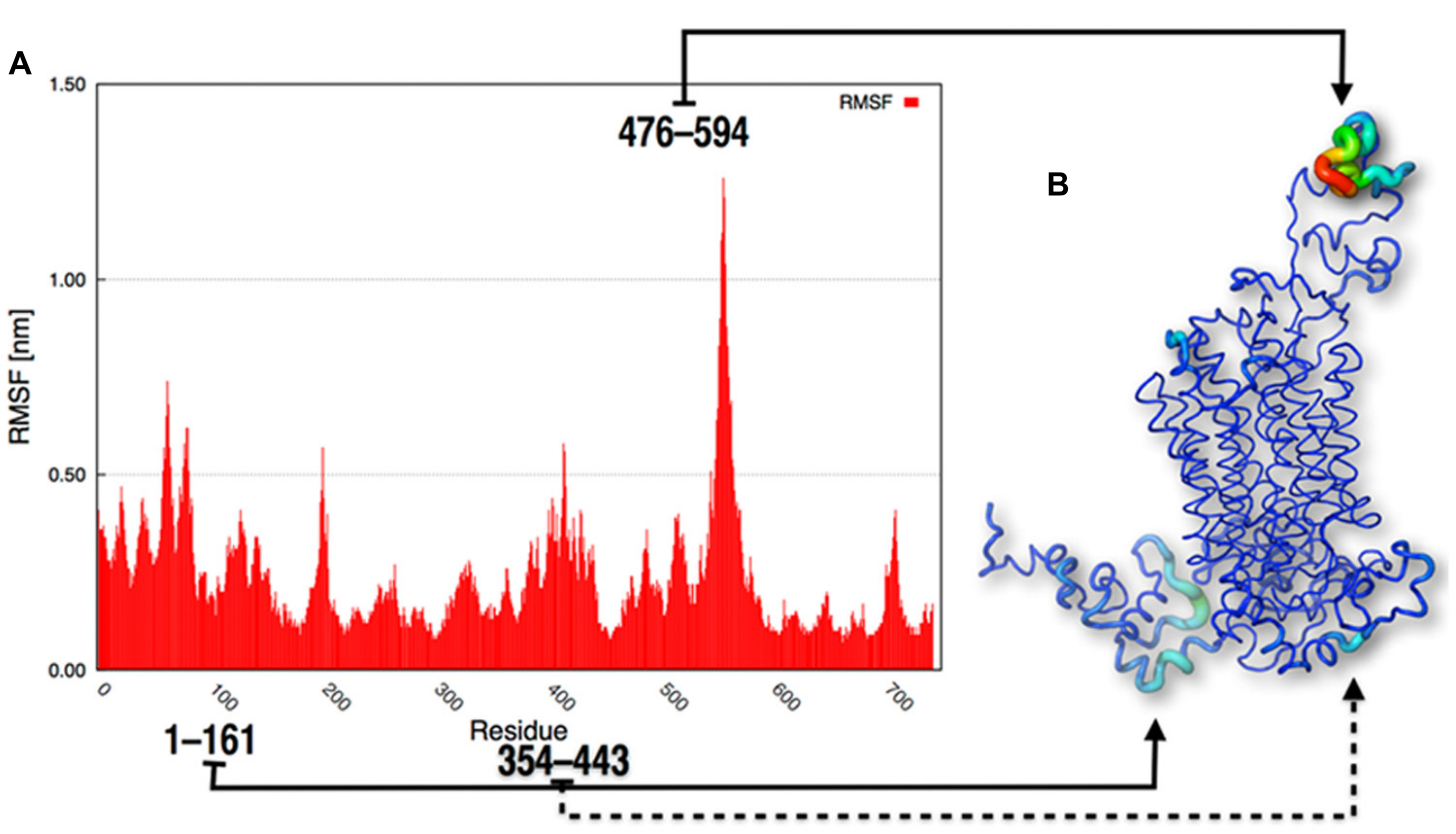
**Graphic RMSF representations. (A)** RMSF of the atomic positions and **(B)** representation of protein flexibility as a gradient from low to high fluctuations.

To obtain more structural details about the behavior of the protein, the RMSF of the α-carbon positions throughout the whole trajectory were calculated. These values indicated that the most flexible parts of SV2A were the loops located outside the membrane; i.e., the N-terminus, the large cytoplasmic loop between the sixth-seventh TM regions and the large intra-luminal loop between the seventh-eighth TM domains (residues 476–594; **Figure 6**).

**FIGURE 7:**
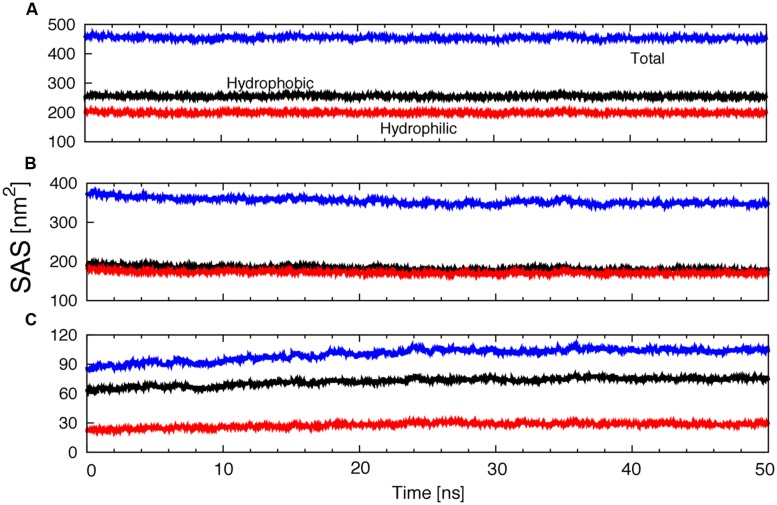
**Evolution of the total, hydrophobic and hydrophilic protein SAS; **(A)** exposed to water plus lipids, **(B)** exposed to water or **(C)** exposed to lipids as a function of time**.

On the other hand, protein solvent contacts were studied using SAS, which is a measure of the protein surface exposed to the solvent. The total SAS and the contributions of hydrophobic or hydrophilic groups to the SAS were determined as a function of time (**Figure 7A**). No significant changes were observed along the trajectory. The SAS of the protein exposed to water decreased slightly (**Figure 7B**), whereas the SAS of the protein exposed to lipids increased a little (**Figure 7C**).

**FIGURE 8:**
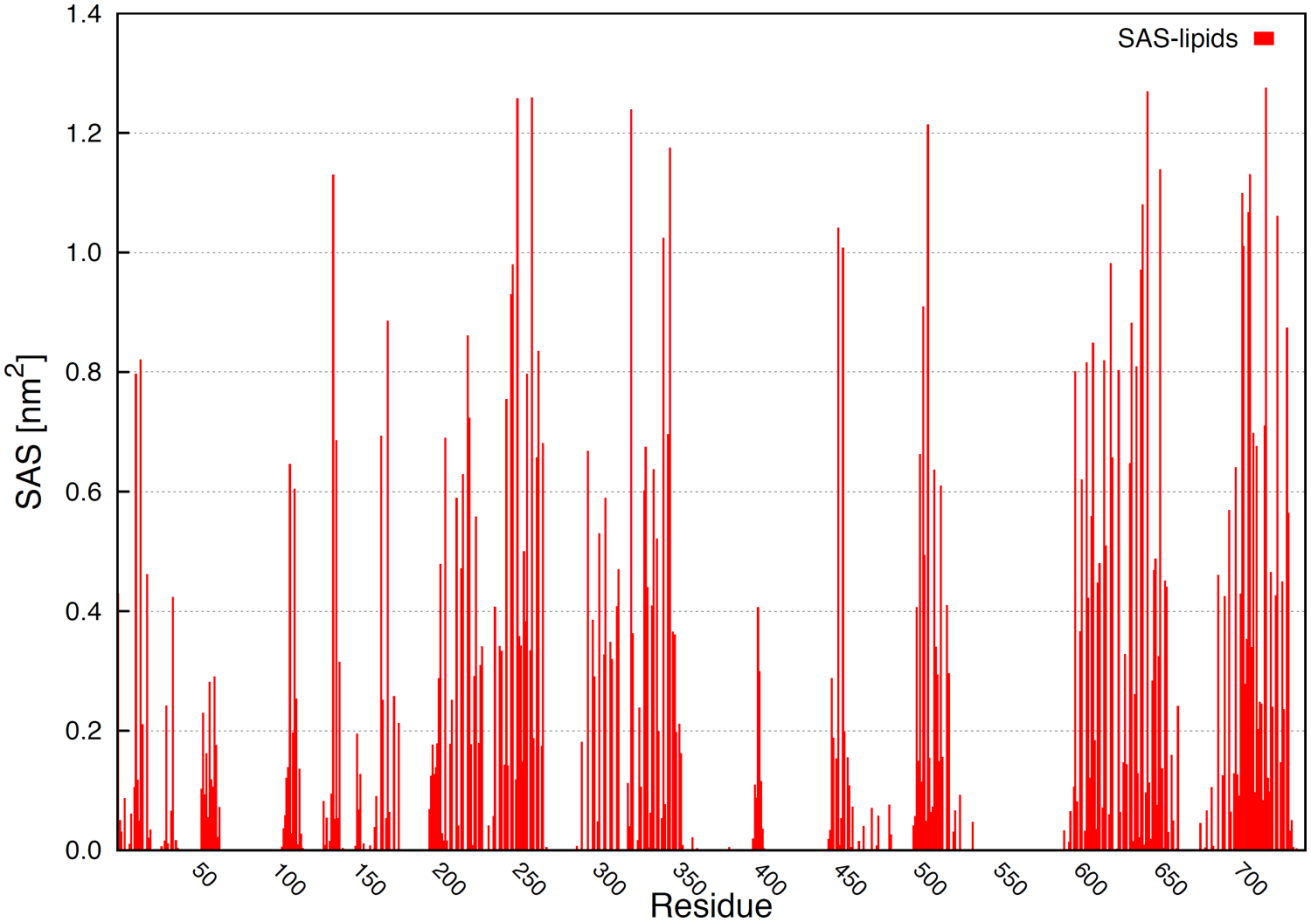
**Solvent accessible surface area by residues exposed to the membrane inner core.** The highest values correspond to amino acids totally exposed to membrane hydrophobic core.

Solvent accessible surface area by residues exposed to the membrane hydrophobic core helps to evidence the localization of several amino acids. **Figure 8** shows the total area exposed to the membrane by residues. Some residues in the loops, N-terminus and ATP binding motif interacted with the membrane. The TM helical domains can be determined, since the highest SAS values correspond to residues totally exposed to the membrane hydrophobic core (**Figure 8**).

**FIGURE 9:**
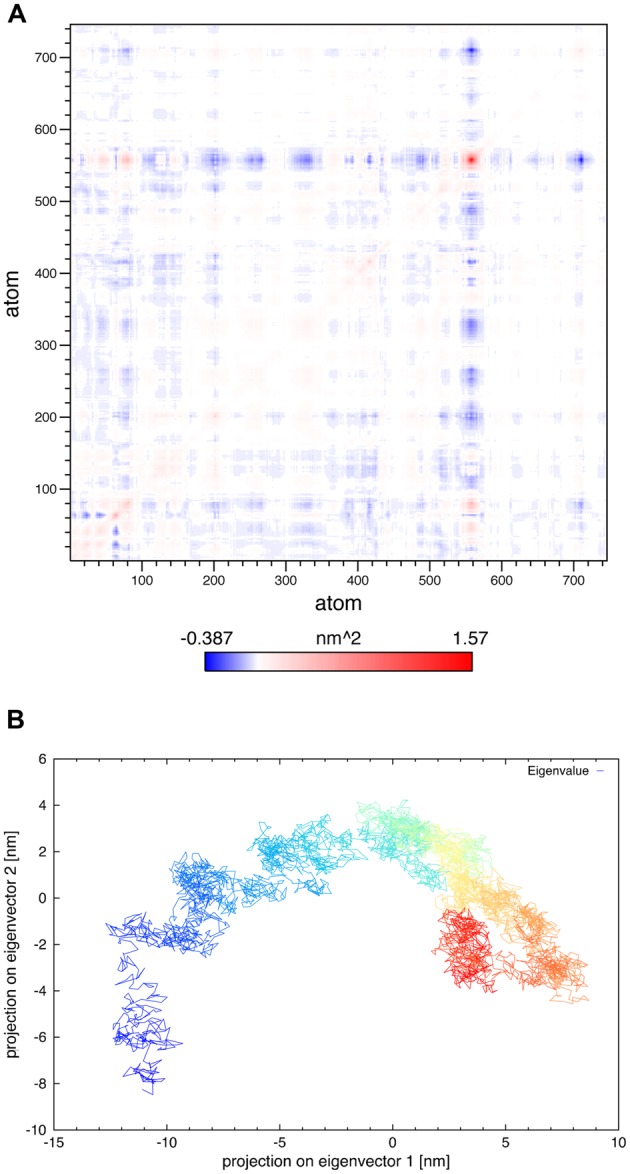
**PCA analysis. (A)** Variance–covariance matrix of the SV2A α-carbon atoms for the 50 ns of the MDS. **(B)** Projection of the 50 ns trajectory onto the two principal eigenvectors obtained from the diagonalization of variance–covariance matrix; time along the trajectory is represented by a denser gradient.

In principal components analysis, the α-carbon matrix of SV2A shows a positive correlation between atomic motions of the large extra-vesicular loop and N-terminal segment (**Figure 9A**). It revealed that only 2–3 components are relevant for the trajectory. The projection of the trajectory onto the two principal eigenvectors shows a structure that reached equilibrium in the last portion of the trajectory; illustrated by denser yellow–red regions (**Figure 9B**).

The results of the replicate MDS were, in general, in agreement with the data obtained in the first MDS (data not shown). Although details varied, all MDS parameters behaved in a similar manner. In particular, the TM domains arrived at a conformational equilibrium; whereas the extra-membrane components remained mobile.

### Docking Studies

The values for the ΔG of binding between SV2A and the racetams varied between -5.26 and -8.82 Kcal/mol throughout the last 16 ns of the MDS trajectory (**Table 1**). The results showed the same affinity pattern in all evaluated conformations: UCB30889 > BRIV > SEL > LEV; which is in agreement with experimental SV2A binding data (**Table 2**; Noyer et al., 1995; Gillard et al., 2003, 2011; Bennett et al., 2007).

**Table 1:** Free energy of binding and residues involved in the interaction between the racetam derivatives and nine SV2A conformers during the MD trajectory (corresponding to every 2 ns during the last 16 ns), bold residues interacted most frequently.

SV2Asnapshot	Ligand	ΔG(Kcal/mol)	Residues
34 ns	LEV	-5.88	G452, V453, F455, **T456**, L628, A664, **S665**, **W666**, A668, L669, **D670**, **L689**, C693
	BRIV	-6.58	I273, V453, **T456**, Y461, Y462, A664, **S665**, **W666**, N667, A668, L669, **D670**, F686, **L689**, N690, C693
	SEL	-6.06	G452, V453, F455, **T456**, Y462, L628, A664, **S665**, **W666**, L669, **D670**, F686, **L689**, N690, C693
	UCB30889	-8.72	D179, I273, M449, M450, G452, V453, F455, **T456**, Y461, Y462, L628, A664, **S665**, **W666**, L669, **D670**, **L689**, N690, C693
36 ns	LEV	-5.49	I273, V453, **T456**, Y461, Y462, **S665**, **W666**, N667, **D670**, F686, **L689**, N690, C693, K694
	BRIV	-6.28	D179, V183, I273, W300, **T456**, Y461, Y462, **S665**, **W666**, N667, **D670**, F686, **L689**, N690, C693, K694, L695
	SEL	-5.83	I273, G452, V453, F455, **T456**, Y461, Y462, A664, **S665**, **W666**, N667, L669, **D670**, F686, **L689**, N690, C693, K694
	UCB30889	-8.8	I273, G452, V453, **T456**, Y461, Y462, L628, A664, **S665**, **W666**, A668, L669, **D670**, F686, **L689**, N690, C693, K694
38 ns	LEV	-5.26	M449, G452, V453, F455, **T456**, F459, L628, S632, A664, **S665**, **W666**, A668, L669, **D670**, **L689**
	BRIV	-6.16	D179, I273, W300, **T456**, Y461, Y462, A664, **S665**, **W666**, **D670**, F686, **L689**, N690, C693
	SEL	-5.5	V183, G205, G208, L209, Y212, **T456**, M457, S458, S460, Y461, Y462, G463, A697, I701
	UCB30889	-8.36	V183, V186, K204, G205, G208, L209, Y212, **T456**, M457, S460, Y461, Y462, V466, A697, I701
40 ns	LEV	-5.42	D179, V183, I273, W300, **T456**, Y461, Y462, **S665**, **W666**, F686, N690, C693, K694
	BRIV	-6.4	D179, I273, W300, Y461, Y462, **S665**, **W666**, **D670**, F686, **L689**, N690, K694, C693
	SEL	-5.9	D179, V183, I273, W300, Y461,Y462, **S665**, **W666**, **D670**, F686, **L689**, N690, C693, K694
	UCB30889	-8.25	I273, G452, V453, F455, **T456**, F459, Y461, Y462, A664, **S665**, **W666**, L669, **D670**, F686, **L689**, N690, C693, K694
42 ns	LEV	-5.67	G452, V453, F455, **T456**, L628, A664, **S665**, **W666**, N667, A668, L669, **D670**, V671, **L689**
	BRIV	-6.25	G452, F455, V453, **T456**, Y462, A664, **S665**, **W666**, L669, **D670**, F686, **L689**, N690, C693
	SEL	-5.81	G452, V453, F455, **T456**, Y461, Y462, L628, A664, **S665**, **W666**, A668, L669, **D670**, F686, **L689**, N690, C693
	UCB30889	-8.49	I273, G452, V453, F455, **T456**, Y461, Y462, L628, A664, **S665**, **W666**, A668, L669, **D670**, F686, **L689**, N690, C693
44 ns	LEV	-5.67	L176, D179, V183, Y212, I273, F277, W300, Y461, Y462, **S665**, **W666**, N690, K694
	BRIV	-6.91	L176, D179, V183, Y212, I273, F277, W300, Y461, Y462, **S665**, **W666**, N667, **D670**, F686, N690, K694
	SEL	-6.43	L176, D179, V183, Y212, I273, F277, W300, Y461, Y462, **S665**, **W666**, N667, **D670**, N690, K694
	UCB30889	-7.82	L176, D179, V183, Y212, I273, F277, W300, V453, **T456**, Y461, Y462, **S665**, **W666**, L669, **D670**, F686, **L689**, N690, C693, K694
46 ns	LEV	-5.95	M449, M450, G452, V453, F455, **T456**, L628, A664, **S665**, A668, L669, **D670**, **L689**, C693
	BRIV	-7.01	M449, M450, G452, V453, F455, **T456**, F459, L628, A664, **S665**, A668, L669, **D670**, **L689**, C693
	SEL	-6.88	M449, M450, G452, V453, F455, **T456**, F459, L628, A664, **S665**, A668, L669, **D670**, **L689**, C693
	UCB30889	-8.51	E182, V183, V186, K204, G205, L207, G208, L209, V211, Y212, R262, Y462, G463, V466
48 ns	LEV	-5.73	M449, G452, V453, F455, **T456**, L628, A664, **S665**, A668, L669, **D670**, **L689**, C693
	BRIV	-6.24	L176, D179, I273, F277, W300, Y461, Y462, **S665**, **W666**, **D670**, F686, N690, K694
	SEL	-6.04	M449, G452, V453, W454, F455, **T456**, L628, A664, **S665**, A668, L669, **D670**, F686, **L689**, N690, C693
	UCB30889	-8.82	I273, F277, G452, V453, F455, **T456**, F459, Y461, Y462, A664, **S665**, **W666**, L669, **D670**, F686, **L689**, N690, C693, K694
50 ns	LEV	-5.33	L176, D179, I273, F277, W300, **T456**, Y461, Y462, **S665**, **W666**, **D670**, F686, N690, K694
	BRIV	-6.67	F277, G452, V453, F455, **T456**, Y461, A664, **S665**, **W666**, N667, L669, **D670**, F686, **L689**, N690, C693
	SEL	-6.29	F277, G452, V453, F455, **T456**, Y461, **S665**, **W666**, N667, L669, **D670**, F686, **L689**, N690, C693
	UCB30889	-8.33	I273, F277, G452, V453, F455, **T456**, Y461, Y462, A664, **S665**, **W666**, L669, **D670**, F686, **L689**, N690, C693, K694


**Table 2:** Mean ΔG of binding during the MD trajectory and experimental SV2A binding data (see text for references).

Ligand	ΔG (kcal/mol, Mean ± SD)	Experimental SV2A pK_d_
Levetiracetam	-5.60 ± 0.24	6.1
Brivaracetam	-6.50 ± 0.31	7.2
Seletracetam	-6.08 ± 0.41	7.1
UCB30889	-8.46 ± 0.32	7.6

**FIGURE 10:**
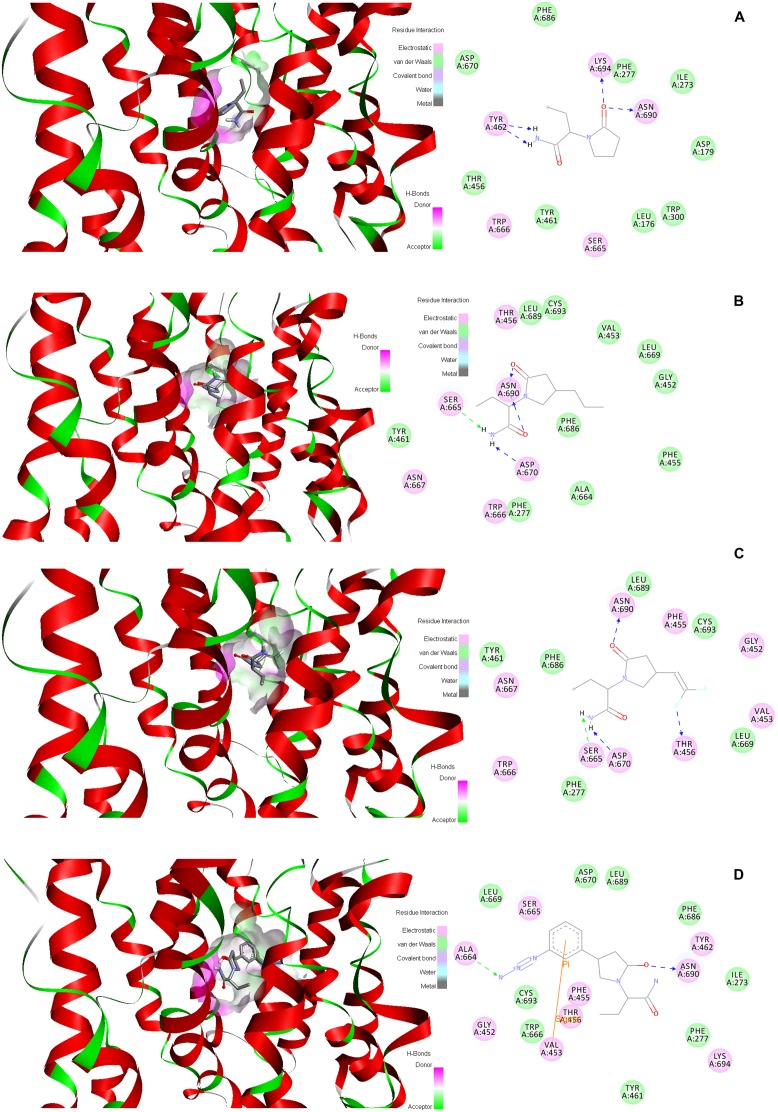
**Putative racetam binding site within the last SV2A conformer of the MDS.** 3D models and schematic representations of the interactions between SV2A and **(A)** LEV, **(B)** BRIV,** (C)** SEL or **(D)** UCB30889 are shown. Hydrophobic interactions and H-bonds participate importantly in ligand recognition within the binding site (amino acids in circles).

Ligand binding in dockings showed a clear preference for the following residues: T456, S665, W666, D670 and L689. These residues are within TM domains 7, 10 and 11. According to the SAS analysis these amino acids show values of zero or very close, which means that they are totally exposed to the membrane hydrophilic core (i.e., the pore or channel). Contacts between SV2A and the racetams were dominated by hydrophobic interactions and H-bonds. Every racetam showed a particular pattern of interactions when bound to SV2A; even if they shared some residues, the interaction was not the same in all cases. **Figure 10** shows schematic representations of the interactions between the last SV2A conformer (50 ns) of the MDS and LEV (**Figure 10A**), BRIV (**Figure 10B**), SEL (**Figure 10C**) or UCB30889 (**Figure 10D**).

## Discussion

Trans-membrane proteins are difficult to crystallize; thus computational techniques such as protein folding using homology modeling and MDS have been very useful tools to obtain information about their structural properties. The I-Tasser server was used to generate a full-length 3D model of SV2A with adequate structural predictions and acceptable quantitative assessments (Zhang and Skolnick, 2004; Zhang, 2008; Nugent and Jones, 2012). Modeling the extra-membrane loops was much more challenging than the TM regions, primarily due to sparse contacts or poor predictions of them; additionally, inherent loop flexibility imposed extra difficulties. Nevertheless, I-Tasser modeled the N-terminus and the two long loops quite similar to the secondary structure predicted by UniProt (2014). It is clear, however, that more sophisticated strategies will be required to overcome a number of current limitations in this area (Nugent and Jones, 2012).

Although the obtained structure was acceptable, it was further refined by running a MDS. It showed the time dependent behavior of SV2A and provided detailed information on its fluctuations and conformational changes. The RMSD results showed that SV2A reached equilibrium in the last 15 ns of the MDS and this was in agreement with the Rg values. This was also true for the number of H-bonds, since they remained constant during the last part of the MDS. The results showed that the TM domains reached equilibrium and were likely to be correctly modeled; whereas the regions outside the membrane still remained mobile (also shown by the RMSF values). This is a general occurrence for TM proteins (Nugent and Jones, 2012) and it has been reported for other proteins, such as Bcl-2 (Ilizaliturri-Flores et al., 2013).

Extra-membrane domains, such as the long intra-luminal loops and N-terminus of SV2A, have post-translational modifications and interactions with other molecules, which might stabilize the protein conformation in natural conditions (Schivell et al., 1996, 2005; Nowack et al., 2010). The lack of post-translational changes and contacts with other molecules (which were not modeled due to their added complexity) may contribute to the extensive loop motion. Moreover, the extra-membrane regions of SV2A were evocative of intrinsically disordered domains within other proteins, such as Bcl-2 (Rautureau et al., 2010). All of the above, plus difficulties associated to proteins embedded in the lipid bilayer, may explain why SV2A has not been crystallized (Hipolito et al., 2014). The relevance of post-translational modifications on the conformational stability could be explored in future works with new, longer MD simulations. It is important to note that the racetam binding site is not part of the extra-membrane regions and is within the pore, a region in equilibrium for at least the last 15 ns of the MDS; thus, our model proves to be of acceptable quality.

Synaptic vesicle protein 2A underwent amino acids rearrangements, mostly in the loop structure components. **Figure 2** and the SAS analysis showed an increase in the protein portion exposed to lipids; suggesting that amino acids initially exposed to the aqueous solvent were moving into the lipid bilayer. It is likely that they required a more hydrophobic environment or more favorable interactions with the lipid heads. In principal components analysis, the variance–covariance matrix of atomic motions showed which atoms moved together in a concerted way. It confirmed the conformational change that occurred in the large extra-vesicular loop and N-terminus, which were the most difficult regions to model and also the most flexible parts of the protein. The projection of the trajectory onto the two principal eigenvectors indicated that the protein experienced serious structural evolution during the first 15 ns of the MDS; however, its structure reached equilibrium in the last part of the trajectory.

We performed docking studies to explore the recognition pattern of racetams in SV2A. Docking studies can be performed on protein structures obtained from homology modeling, which maintains the backbone conformation (Tovchigrechko et al., 2002). However, the SV2A 3D structure obtained from I-Tasser was refined by performing a MDS; the advantage was that the side chain residues were energetically accommodated to their neighbors, resulting in several different conformers. Since the docking procedure lacks protein flexibility, we used the protein conformers from the MDS to obtain more reliable data, as has been published elsewhere (Sixto-Lopez et al., 2014). The MDS and docking simulations were thus combined, since using only one conformation may omit several structural properties and result in non-biological dockings (Ramirez-Duran et al., 2013). Our docking studies suggested a consensus binding site for racetam ligands within SV2A constituted by five residues: T456, S665, W666, D670 and L689. Tryptophan 666 directly engages the racetams, mostly by electrostatic interactions. The SV2A protein with a mutated W666 did not restore synaptic depression in neurons lacking SV2A and SV2B; showing that this residue is vital for SV2A neurotransmitter function (Nowack et al., 2010). Additionally, binding of UCB30889 to SV2A is altered when this amino acid is mutated, indicating that it may participate in ligand recognition (Shi et al., 2011).

The SV2A protein has been proposed to be a transporter owing to its high degree of homology with other mayor facilitator super-family (MFS) carriers (Bajjalieh et al., 1992; Feany et al., 1992; Gingrich et al., 1992) and its compact funnel-shaped structure with a visible indentation in the center indicative of a pore opening (Lynch et al., 2008). Tryptophan 666 is conserved in all SV2 isoforms and is homologous to a tryptophan in the 10th TM domain of MFS transporters which is vital for their activity (Nowack et al., 2010; Shi et al., 2011). Thus, it may provide the necessary hydrophobic milieu for transport of an endogenous substrate or LEV, in addition to its involvement in racetam binding. Residues Y461 and Y462 also frequently interacted with the racetams (**Table 1**); both are homologous to functional amino acids of MFS transporters (Shi et al., 2011). Additionally, Y461 is homologous to a residue important for chloride carrier activity (Bostick and Berkowitz, 2004).

It has been shown that 14 TM residues alter binding of UCB-30889 to SV2A when mutated (Shi et al., 2011). The present results coincide in the importance of W666 for ligand binding. The other four residues reported here were not mutated in the previous study. Y462 was also repeatedly found in this study; however, the 12 other amino acids described in the prior work were not regularly detected. These discrepancies may be due to the following: (1) SV2A was transiently expressed in COS-7 cells which are far removed from neurons, (2) the true functionality of SV2A mutants was unclear since they were not likely to exit the endoplasmic reticulum or Golgi (Nowack et al., 2010), (3) the residues that altered binding to UCB-30889 were not necessarily part of the ligand pocket; they may affect other aspects, such as changing the native conformation or stability of the protein. This, in turn, may change important aspects, such racetam access, retention or expulsion. These phenomena are not modeled by docking studies, which only predict ligand affinity for a region of the protein. A MDS of SV2A together with the racetams would be useful to further refine the interactions of SV2A and its racetam ligands.

The 3D model generated by I-Tasser and the structure obtained after the MDS show that the TM portions of SV2A may be accessed by the racetams. As previously suggested, LEV and related racetams likely enter into recycling vesicles, gain access to SV2A from its luminal side and bind in its TM segments (Meehan et al., 2011, 2012; Mendoza-Torreblanca et al., 2013). Once bound to SV2A, racetams inhibit its function; reducing the ready releasable vesicular pool and synaptic transmission, preferentially during long high frequency activity (Xu and Bajjalieh, 2001).

Modeling of the complete SV2A protein was challenging due to its large size (742 amino acids), its long N-terminus and its two long loops. However, a valid 3D model was obtained and the 50 ns of MDS were enough to reach structural equilibrium of the protein. The racetam binding site in SV2A was identified by docking studies employing different SV2A snapshots to explore the protein motion involved in ligand recognition; it showed that five residues participate in ligand recognition. Given the biological and pharmacological importance of SV2A, knowing its conformation is essential to direct further experimental work in order to elucidate its function and attempt the discovery of new ligands. Additionally, knowing the racetam binding site within SV2A should facilitate synthesizing suitable radio-ligands for PET studies with a high affinity and specificity. This, in turn, should allow the *in vivo* evaluation of treatment response and possibly disease progression.

## Author Contributions

All authors contributed to the written manuscript. All authors have given approval to the final version of the manuscript.

## Conflict of Interest Statement

The authors declare that the research was conducted in the absence of any commercial or financial relationships that could be construed as a potential conflict of interest.

## Abbreviations

AM1: Austin Model 1
BRIV: brivaracetam
CNS: central nervous system
H-bonds: hydrogen bonds
ΔG: free energy
LEV: levetiracetam
MFS: mayor facilitator family
MDS: molecular dynamics simulation
OPLS-AA: optimized potentials for liquid simulations all atoms
PET: positron emission tomography
POPC: 1-palmitoyl-2-oleoyl-sn-glycero-3-phosphocholine
Rg: radius of gyration
RMSD: root mean square deviation
RMSF: root mean square fluctuations
SAS: solvent accessible surface area
SD: standard deviation
SEL: seletracetam
SV2A: synaptic vesicle protein 2A
3D: three-dimensional
TM: trans-membrane
Uniprot: universal protein resource
VMD: visual molecular dynamics.
